# Do physical activity and screen time mediate the association between European fathers’ and their children’s weight status? Cross-sectional data from the Feel4Diabetes-study

**DOI:** 10.1186/s12966-019-0864-8

**Published:** 2019-11-04

**Authors:** Julie Latomme, Nele Huys, Greet Cardon, Philip J. Morgan, Mina Lateva, Nevena Chakarova, Jemina Kivelä, Jaana Lindström, Odysseas Androutsos, Esther M. González-Gil, Pilar De Miguel-Etayo, Anna Nánási, László R. Kolozsvári, Yannis Manios, Marieke De Craemer, on behalf of the Feel4Diabetes-study group, Yannis Manios, Meropi Kontogianni, Odysseas Androutsos, George Moschonis, Konstantina Tsoutsoulopoulou, Christina Mavrogianni, Christina Katsarou, Eva Karaglani, Eirini Efstathopoulou, Ioanna Kechribari, Konstantina Maragkopoulou, Effie Argyri, Athanasios Douligeris, Mary Nikolaou, Eleni-Anna Vampouli, Katerina Kouroupaki, Roula Koutsi, Elina Tzormpatzaki, Eirini Manou, Panagiota Mpinou, Alexandra Karachaliou, Christina Filippou, Amalia Filippou, Jaana Lindström, Tiina Laatikainen, Katja Wikström, Karoliina Nelimarkka, Jemina Kivelä, Päivi Valve, Greet Cardon, Julie Latomme, Vicky Van Stappen, Nele Huys, Lieven Annemans, Lore Pil, Peter Schwarz, Ivonne Panchyrz, Maxi Holland, Patrick Timpel, Konstantinos Makrilakis, Stavros Liatis, George Dafoulas, Christina-Paulina Lambrinou, Angeliki Giannopoulou, Lydia Tsirigoti, Evi Fappa, Costas Anastasiou, Konstantina Zachari, Lala Rabemananjara, Dimitrios Kakoulis, Mayur Mandalia, Maria Stella de Sabata, Niti Pall, Luis Moreno, Fernando Civeira, Gloria Bueno, Pilar De Miguel-Etayo, M. Esther, Maria I. Mesana, Germán Vicente-Rodriguez, Gerardo Rodriguez, Lucia Baila-Rueda, Ana Cenarro, Estíbaliz Jarauta, Rocío Mateo-Gallego, Violeta Iotova, Tsvetalina Tankova, Natalia Usheva, Kaloyan Tsochev, Nevena Chakarova, Sonya Galcheva, Rumyana Dimova, Yana Bocheva, Zhaneta Radkova, Vanya Marinova, Imre Rurik, Timea Ungvari, Zoltán Jancsó, Anna Nánási, László Kolozsvári, Remberto Martinez, Marcos Tong, Kaisla Joutsenniemi, Katrina Wendel-Mitoraj

**Affiliations:** 1 Department of Movement and Sports Sciences, Ghent University, Ghent, Belgium; 2 School of Education, PRCPAN (Priority Research Centre for Physical Activity and Nutrition), University of Newcastle, Newcastle, Australia; 3 Clinic of Paediatric Endocrinology, Medical University Varna, Varna, Bulgaria; 4 Clinical Center of Endocrinology, Medical University of Sofia, Sofia, Bulgaria; 5 National Institute for Health and Welfare, Helsinki, Finland; 6 School of Health Science & Education, Department of Nutrition and Dietetics, Harokopio University, Athens, Greece; 7 GENUD (Growth, Exercise, Nutrition and Development), University of Zaragoza, Zaragoza, Spain; 8 Department of Family and Occupational Medicine, University of Debrecen, Debrecen, Hungary

**Keywords:** Obesity, Fathers, Children, Intervention, Physical activity, Sedentary behaviour, Screen time behaviour

## Abstract

**Background:**

Most research on parenting and childhood obesity and obesity-related behaviours has focused on mothers while fathers have been underrepresented. Yet, recent literature has suggested that fathers uniquely influence their children’s lifestyle behaviours, and hence could also affect their weight status, but this has not yet been scientifically proven. Therefore, the present study aimed to determine whether the association between fathers’ weight status and their children’s weight status is mediated by fathers’ and children’s movement behaviours (i.e. physical activity (PA) and screen time (ST)).

**Methods:**

Cross-sectional data of 899 European fathers and their children were analyzed. Fathers/male caregivers (*mean age* = 43.79 ± 5.92 years, *mean BMI* = 27.08 ± 3.95) completed a questionnaire assessing their own and their children’s (*mean age* = 8.19 ± 0.99 years, 50.90% boys, *mean BMI*_*zscore*_ = 0.44 ± 1.07) movement behaviours. Body Mass Index (BMI, in kg/m^2^) was calculated based on self-reported (fathers) and objectively measured (children) height and weight. For children, BMI z-scores (SD scores) were calculated to obtain an optimal measure for their weight status. Serial mediation analyses were performed using IBM SPSS 25.0 Statistics for Windows to test whether the association between fathers’ BMI and children’s BMI is mediated by fathers’ PA and children’s PA (model 1) and fathers’ ST and children’s ST (model 2), respectively.

**Results:**

The present study showed a (partial) mediation effect of fathers’ PA and children’s PA (but not father’s ST and children’s ST) on the association between fathers’ BMI and children’s BMI (model for PA; coefficient: 0.001, 95% CI: [0.0001, 0.002]; model for ST; coefficient: 0.001, 95% CI: [0.000, 0.002]). Furthermore, fathers’ movement behaviours (PA and ST) were positively associated with their children’s movement behaviours (PA and ST) (model for PA, coefficient: 0.281, SE: 0.023, *p* < 0.001; model for ST, coefficient: 0.345, SE: 0.025, *p* < 0.001).

**Conclusions:**

These findings indicate that the influence of fathers on their children’s weight status partially occurs through the association between fathers’ PA and children’s PA (but not their ST). As such, intervening by focusing on PA of fathers but preferably of both members of the father-child dyad (e.g. engaging fathers and their children in co-PA) might be a novel and potentially effective strategy for interventions aiming to prevent childhood overweight and obesity. Longitudinal studies or intervention studies confirming these findings are however warranted to make meaningful recommendations for health intervention and policy.

**Trial registration:**

The Feel4Diabetes-study is registered with the clinical trials registry http://clinicaltrials.gov, ID: 643708.

## Background

Childhood overweight and obesity are currently one of the most serious public health concerns as they consistently have been associated with a wide range of negative biological, psychological, and social health consequences [1]. Along with dietary intake, two important behaviours play an important role in the development of overweight and obesity are physical activity (PA) and sedentary behaviour (SB), of which screen time (ST) (e.g. TV viewing and computer use) is the most common form [2]. Unhealthy patterns of these so-called “movement behaviours” [3] can be found in many European primary school-aged children. Recent evidence has for example shown that 4.6 to 16.8% of European primary school-aged children (10–12 years old) does not meet the international recommendation of at least 60 min of moderate-to-vigorous PA per day, and 19.0 to 31.7% (weekdays) and 57.4 to 71.2% (weekend days) of the European primary school-aged children (6–9 years old) exceeds the internationally recommended guideline [3–5] of no more than 2 h recreational ST per day [6]. The establishment of healthy patterns of PA and SB (including ST) during childhood is however important, as they tend to track into adolescence and adulthood [7–9]. As such, targeting these movement behaviours at a young age has become an important focus in health promotion and obesity prevention research [10, 11].

This can be framed within the socio-ecological model of health behaviour, which is a model often used in health research offering a broad perspective on health behaviours, integrating multiple hierarchically-nested levels of influence. According to this theoretical model, influences from the interpersonal level –which is the closest to the child and contains the structures with which the child has direct contact, such as family, school, neighborhood, or childcare environments- are the strongest and have the greatest impact on the child [12, 13]. Within this interpersonal level, it has been widely stated in the literature that parents play a key role in establishing positive health behaviours in their children [14, 15]. However, a large drawback of this assumption is that most of the studies included only mothers, while fathers have been largely underrepresented [16–18]. More specifically, there is a paucity of research on the (specific) impact of fathers on their children’s health behaviours [19]. In a systematic review identifying the inclusion of fathers as research participants in observational studies investigating parental influences on childhood obesity or obesity-related behaviours, it was found that fathers represented only 17% of parents across the 667 eligible studies, 48% of which included no fathers at all [17]. Overall, only 10% of the studies reported father-specific data and only 1% of the studies included only fathers. This is a major evidence gap, as overlooking the (unique) contribution of fathers has reduced our understanding of factors contributing to childhood obesity and hindered the development of effective family-based intervention programs [16]. Moreover, recent research has indicated that the influence of fathers may be important for predicting childhood obesity [20, 21]. Several longitudinal studies have for example shown that weight status of the father is a significant and important predictor of their children’s weight status [20, 21]. Furthermore, some recent studies also showed a positive association between movement behaviours of the father (i.e. PA and SB) and those of their children, which was independent from the mother [22–32]. However, studies examining PA associations are still limited [33]. For SB, the existing evidence is even more scarce and research findings are inconclusive [25, 33, 34].

Most importantly, no studies investigated the interrelationships between fathers’ and children’s weight status and their movement behaviours (i.e. PA and ST). This is important to understand the potential pathways between father and child weight status and it might provide novel intervention modality in the fight against childhood obesity. Therefore, this study aimed to determine whether the association between fathers’ and children’s weight status is mediated by respectively fathers’ and children’s movement behaviours (i.e. PA and ST). We hypothesize that this will indeed be the case, and if so, lifestyle interventions could focus on the father’s movement behaviours in order to prevent childhood obesity, in addition to children’s and mothers’ behaviours. Last, a major shortcoming in research on this topic is that most studies on this topic are national studies (e.g. conducted in Australia, Canada, USA, UK and Portugal), predominantly coming from high-income countries [35]. Given the different occupational and socio-cultural structures, environmental factors (e.g. safety, climate), etc. in middle- to low-income countries, evidence from these countries is needed too [36]. The current study addresses these shortcomings by investigating data from six European countries, representing different socio-economic levels. Additionally, examining large-scale international data also increases the generalizability of the results and allows an examination of associations regardless of the specific characteristics of a country.

## Methods

### Study background and data collection

This study performed secondary data analysis on cross-sectional data from the “Feel4Diabetes-study”, which was conducted in six European countries representing low income countries (Bulgaria and Hungary), high income countries (Belgium and Finland) and countries under austerity measures (Greece and Spain). Recruitment was conducted within the provinces of Oost-Vlaanderen and West-Vlaanderen (Belgium), Varna and Sofia (Bulgaria), Satakunta (Finland), Attica (Greece), Debrecen and its county (Hungary) and Zaragoza (Spain). In Bulgaria and Hungary, all areas within the selected provinces were eligible to participate in Feel4Diabetes. In Greece, Spain, Finland and Belgium, the municipalities, school districts or other equivalent units in the selected provinces were grouped in tertiles according to socio-economic indices retrieved from official resources and authorities areas were randomly selected only from the tertile with the lowest education level or the highest unemployment rate. In the case of Finland, areas were ordered based on the mean values of the selected socioeconomic index and areas were selected from the lower mean. In all countries, after taking the necessary approval(s) from local authorities (ethical committees, ministries, municipalities, etc.), lists of all primary schools within the randomly selected areas were created and primary schools were randomly selected and recruited within each area. Thereafter, children attending the first three grades of compulsory education and their families were then recruited within these schools to participate in the study. More specifically, children received an information letter to take home for their families, in which parents were briefly informed about the purpose of the study. By signing a written informed consent, parents gave permission to participate in the study. All parents/primary caregivers who agreed to participate were then asked to complete a questionnaire, and researchers visited the schools again to objectively measure the weight and height of the participating children (i.e. see the measures section for more information on how this was obtained). More details about this research, data collection and design can be found elsewhere (www.feel4diabetes-study.eu) [37].

### Measures

Within the Feel4Diabetes-study, a questionnaire was developed to be completed (at home) by one of the parents/primary caregivers, who completed this questionnaire both for him/herself and their child. For the present study, only relevant socio-demographics (i.e. fathers’ age, fathers’ education level, and children’s age and sex) and measures on movement behaviours collected with this questionnaire (i.e. PA and ST) were used.

#### Physical activity

Fathers’ and children’s PA were assessed in two questions, i.e. “In the previous week, how many days were you/was your child active for at least 30 min/day (parent)/ 60 min/day (child) (a) on weekdays, and (b) on weekend days? With ‘active’ we mean any kind of movement that makes you sweat a little and increases your heart rate, for example cycling, dancing, gardening, fitness, etc.”. For weekdays, possible answer options varied on a 6-point scale ranging from “none” to “5 days”. For weekend days, possible answer options varied on a 3-point scale ranging from “none” to “2 days”. These categorical values were then recoded into continuous variables (i.e. none was recoded into 0, 1 day was recoded into 1, etc.). The sum of these two variables was used in the analyses as a measure of the fathers’ and children’s amount of PA, reflecting the number of days fathers/children reached the PA guideline.

#### Screen time

Fathers’ and children’s ST were also assessed in two questions, i.e. “How many hours per day do you/does your child spend on screen activities (activities at work/school not included) on (a) weekdays, and (b) on weekend days”. Answer options varied on a 10-point scale, ranging from “none” to “7 or more hours/day”, with a 1 hour range in other options e.g. “2 to less than 3 hours/day”. These categorical variables were recoded into continuous variables using the midpoint method (e.g. “2 to less than 3 h/day” was recoded into 150 min/day, “3 to less than 4 h/day was recoded into 210 min/day) [38], and the average daily amount of the parents’ and children’s ST (min/day) was then calculated using the following formula: ((ST_weekdays_*5) + (ST_weekenddays_*2))/7.

The test-retest reliability of the PA and ST measures was ranked as ‘moderate’ to ‘excellent’ (ICC range = 0.57 to 0.83), except for children’s PA on weekend days and fathers’ ST on weekdays, which was ranked as ‘poor’ (ICC = 0.37 and ICC = 0.33, respectively).

#### Body mass index

Both mothers’ and fathers’ Body Mass Index (BMI, in kg/m^2^) was calculated based on their self-reported weight and height, and children’s BMI was calculated based on their objectively weight and height. More specifically, children were measured at schools by a team of researchers. Height was measured using the Seca 2017 stadiometer for mobile height measurement, and weight was measured using the Seca 813 digital flat scale. For the analyses, BMI z-scores were calculated for the children to obtain an optimal measure for their weight status, relative to their age and sex.

#### Education level

Education level of the father was questioned to have a proxy measure of socio-economic status of the family [39]. This was asked in a 5-point Likert-type scale question, ranging from “less than 7 years” to “more than 16 years” of education.

### Data analysis

**Fig. 1:**
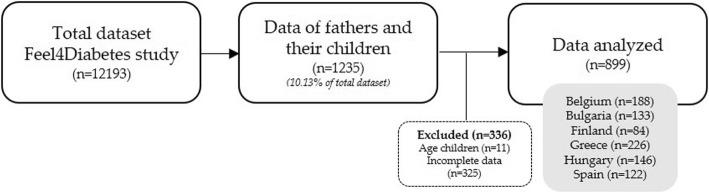
Flow diagram of participants throughout the study

**Fig. 2:**
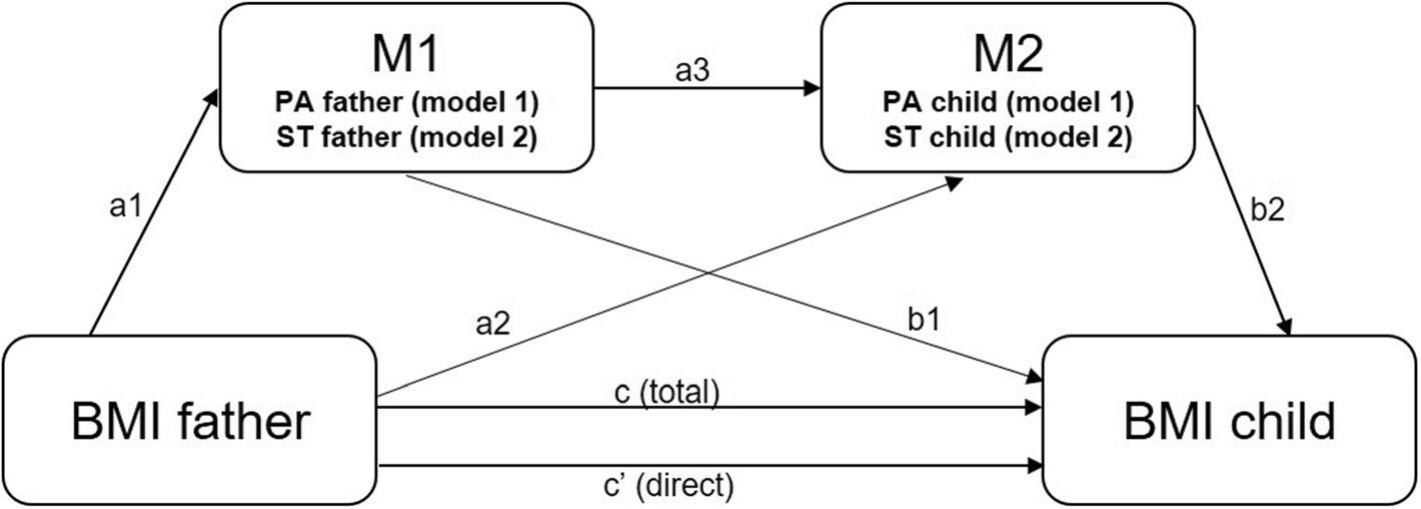
The serial mediation models. Each model with two mediators (M1 and M2) in each model; PA of the father and PA the child in model 1, and ST of the father and ST of the child in model 2. Path a1 represents the effect of BMI of the father on M1, path a2 represents the effect of BMI of the father on M2, path a3 represents the effect of M1 on M2. Path b1 and path b2 represent the effect of respectively M1 and M2 on BMI of the child. Path c represents the total effect of BMI of the father on BMI of the child, and path c’ is the direct effect of BMI of the father on BMI of the child

Data of 899 father-child dyads (i.e. combination of a father/male caregiver with (one) primary school-aged child) were included in the present study. Inclusion criteria were having complete data on the (outcome) variables for both the father and the child and the child’s age between 5 and 13 years old (i.e. primary school age). In total, 325 cases were excluded for not meeting these inclusion criteria, see Fig. 1 for a more detailed description. Descriptive statistics were computed to describe the sample characteristics, using IBM SPSS Statistics for Windows, version 25.0 [40]. As recommended by Baron and Kenny [41] the following assumptions must be fulfilled to establish a mediation effect: i) the predictor and outcome variable need to be significantly correlated, ii) mediators need to be significantly correlated with both the predictor and outcome variable in order to include them in the model. To check these assumptions, Pearson correlation analysis was performed using IBM SPSS Statistics for Windows, version 25.0 [40] between all the proposed mediators (i.e. PA of the father, PA of the child, ST of the father and ST of the child), the predictor (BMI of the father) and the outcome variable (BMI of the child). Using model 6 (i.e. serial mediation model with two mediators) of an SPSS macro provided by Preacher and Hayes (2008) [42], two serial mediation analyses with two mediators each (M1 and M2; see Fig. 2 for a graphical representation) were performed to investigate whether the association between BMI of the father and BMI of the child is mediated by respectively PA of the father and PA of the child (model 1) and by respectively ST of the father and ST of the child (model 2). A mediation effect occurs when the (direct) effect of BMI of the father on BMI of the child is eliminated (complete mediation) or reduced (partial mediation) when controlled for the mediators (M1 and M2). As represented in Fig. 2, ten effects can be estimated in each model [1]; the total effect (c-path), representing the effect of BMI of the father on BMI of the child, [2] the direct effect (c’-path), representing the direct effect of BMI of the father on BMI of the child, [3] five ‘intermediate’ effects; the effect of BMI of the father on M1 (PA or ST of the father) (a1-path), the effect of BMI of the father on M2 (PA or ST of the child) (a2-path), the effect of M1 (PA or ST of the father) on M2 (PA or ST of the child) (a3-path), the effect of M1 (PA or ST of the child) on BMI of the child (b1-path) and the effect of M2 (PA or ST of the child) on BMI of the child (b2-path), and [4] three indirect effects. The total effect (c-path) of BMI of the father on BMI of the child was quantified as the sum of the direct effect (c’-path) and the indirect effects. Three indirect effects of BMI of the father on BMI of the child could be estimated; one through M1 (path a1xb1), one through M2 (path a2xb2), and one through both M1 and M2 (path M1&M2, quantified as the subtraction of the direct effect (c’) and indirect effects via (only) M1 and (only) M2 from the total effect (c) (i.e. c-[c’ + (a1xb1) + (a2xb2)]). To test the mediation effect, 5000 bootstrapped resamples and a 95% confidence interval (CI) were applied to construct the indirect paths. Bias-corrected CI that did not include 0 were considered significant. As an effect size, the completely standardized indirect effect size (effect size_cz_) was calculated, indicating that the outcome variable (BMI of the child) is expected to decrease by the magnitude of effect size standard deviations for every standard deviation increase in the predictor (BMI of the father) indirectly through the mediators M1 and M2. An effect size_cz_ of 0.01 was considered as small, 0.09 as moderate and 0.25 as strong [43]. Fathers’ and children’s age, children’s sex and BMI of the mother were included as covariates in the mediation model to control for their potential confounding effect.

**Fig. 3:**
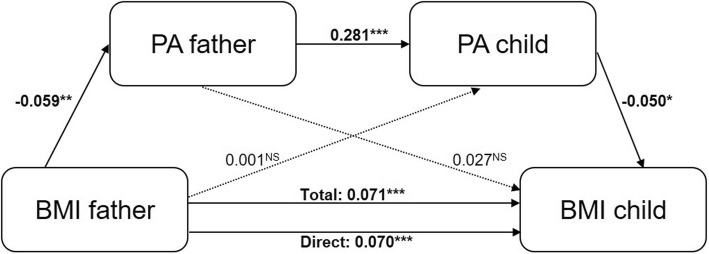
Model 1 of the serial mediation analysis. The association between BMI of the father and BMI of the child through respectively PA of the father (M1) and PA of the child (M2), with each pathway in the serial mediation model. Each arrow with a solid line represents a significant path between variables, an arrow with a dashed line represents a non-significant path. The estimated coefficients are unstandardized. *Note.* *p-value is significant at the 0.05 level, **p-value is significant at the 0.01 level, ***p-value is significant at the 0.001 level, ^NS^non-significant *p*-value

**Fig. 4:**
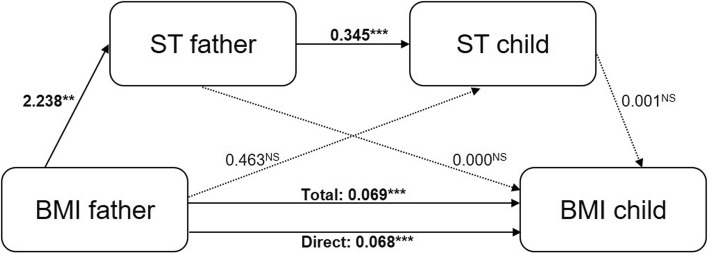
Model 2 of the serial mediation analyses. The association between BMI of the father and BMI of the child through respectively ST of the father (M1) and ST of the child (M2), with each pathway in the serial mediation model. Each arrow with a solid line represents a significant path between variables, an arrow with a dashed line represents a non-significant path. The estimated coefficients are unstandardized. *Note.* *p-value is significant at the 0.05 level, **p-value is significant at the 0.01 level, ***p-value is significant at the 0.001 level, ^NS^non-significant *p*-value

## Results

### Descriptives statistics

In total, data of 899 father-child dyads were analyzed (mean age fathers/male caregivers: 43.79 ± 5.92 years, mean age primary school aged children: 8.19 ± 0.99 years; 50.90% boys). The flow diagram of participants throughout the study can be found in Fig. 1. Descriptive statistics of the sample and variables can be found in Table 1.

**Table 1:** Descriptive statistics

*N* = 899 fathers and children	Fathers	Children
Age (in years)	43.79 (5.92)	8.19 (0.99)
Sex (% male)	100%	50.90%
Education level (% high education^a^)	66.9%	N/A
BMI (in kg/m^2^) *BMI z-scores (children)*	27.08 (3.95)	16.98 (2.73) *0.44 (1.07)*
PA (days per week reaching the PA guideline)	4.39 (2.23)	5.29 (1.63)
ST (minutes screen time activities per day)	134.47 (85.36)	112.59 (69.97)

*Note.* This table provides mean (SD) for the continuous variables and frequency (%) for the categorical variables ^a^13–14 years of education or more

### Correlation analysis

The bivariate correlation analysis showed a significant correlation between the predictor (BMI of the father) and outcome variable (BMI of the child), which was required for testing a mediational effect. Furthermore, all the mediators (i.e. PA of the father, PA of the child, ST of the father and ST of the child) were significantly correlated with both the predictor (BMI of the father) and outcome variable (BMI of the child), and could therefore be included in the mediation models. Last, all proposed covariates were significantly correlated with (one of) the outcome variables, justifying its inclusion in the mediation models. The bivariate correlation analysis results can be found in Table 2.

**Table 2:** Bivariate correlations among fathers’ and children’s PA, ST and BMI

	PA father	PA child	ST father	ST child	BMI father	BMI child
PA child	0.386^c^					
ST father	−0.004	− 0.008				
ST child	0.049	−0.035	0.421^c^			
BMI father	−0.116^c^	−0.048	− 0.084^b^	0.070^a^		
BMI child	−0.013	−0.075^a^	0.030	0.074^a^	0.285^c^	
*Mother BMI*	*−0.049*	*−0.033*	*− 0.101* ^b^	*0.028*	*0.157* ^c^	*0.220* ^c^
*Father age*	*−0.105* ^b^	*−0.038*	*− 0.032*	*0.024*	*0.054*	*−0.009*
*Child age*	*0.100* ^b^	*0.035*	*0.046*	*0.131* ^c^	*−0.015*	*−0.042*
*Child sex*	*−0.031*	*−0.079* ^a^	*0.005*	*−0.023*	*0.038*	*0.035*

*Note.* ^a^correlation is significant at the 0.05 level, ^b^correlation is significant at the 0.01 level, ^c^correlation is significant at the 0.001 level

**Table 3:** Multiple mediation effects through PA (model 1) and ST (model 2) of the father and the child in the association between BMI of the father and BMI of the child

	Coefficient^#^ (SE)	CI upper	CI lower
Model 1 (PA)			
*Intermediate effects*			
a1-path	-0.059 (0.019)**	−0.096	− 0.022
a2-path	0.001 (0.013)	−0.025	0.026
a3-path	0.281 (0.023)***	0.236	0.326
b1-path	0.027 (0.017)	−0.006	0.059
b2-path	−0.50 (0.026)*	−0.094	− 0.006
* Total and direct effect*			
c-path (total effect)	0.071 (0.009)***	0.053	0.086
c’-path (direct effect)	0.070 (0.009)***	0.053	0.087
* Indirect (mediational) effects*			
a1xb1 path (indirect effect via M1)	0.000 (0.001)	−0.004	0.000
a2xb2 path (indirect effect via M2)	0.000 (0.001)	−0.002	0.002
M1&M2 path (indirect effect via M1 and M2)	0.001 (0.001)^+^	0.001	0.002
Model 2 (ST)			
*Intermediate effects*			
a1-path	2.238 (0.725)**	0.816	3.660
a2-path	0.463 (0.542)	−0.600	1.526
a3-path	0.345 (0.025)***	0.296	0.394
b1-path	0.000 (0.001)	−0.001	0.001
b2-path	0.001 (0.001)**	0.000	0.002
*Total and direct effect*			
c-path (total effect)	0.069 (0.009)***	0.053	0.086
c’-path (direct effect)	0.068 (0.009)***	0.051	0.085
*Indirect (mediational) effects*			
a1xb1 path (indirect effect via M1)	0.000 (0.001)	−0.002	0.002
a2xb2 path (indirect effect via M2)	0.000 (0.001)	−0.001	0.002
M1&M2 path (indirect effect via M1 and M2)	0.001 (0.001)^+^	0.000	0.002

*Note.* ^*#*^unstandardized coefficients*,* SE; standard error, CI; confidence interval*,* *p-value is significant at the 0.05 level, **p-value is significant at the 0.01 level, ***p-value is significant at the 0.001 level, ^+^significant indirect effect

### Mediation analysis

Fig. 3 (PA, model 1) and Fig. 4 (ST, model 2) show the association between BMI of the father and BMI of the child, with each pathway in the multiple mediation model. For a detailed description of the results, see Table 3.

#### Total effect and direct effect

The mediation analysis showed a significant total effect (c-path) of BMI of the father on BMI of the child in both models (model 1 (PA), coefficient: 0.071, SE: 0.009, *p* < 0.001; model 2 (ST), coefficient: 0.069, SE: 0.009, *p* < 0.001), indicating that a higher BMI of the father was associated with a higher BMI of the child. Moreover, this effect reduced but remained significant after controlling for the mediators (M1 and M2) in both models (c’, direct effect; model 1 (PA), coefficient: 0.070, SE: 0.009, p < 0.001; model 2 (ST), coefficient: 0.068, SE: 0.009, *p* < 0.001), which indicates a partial mediation effect of the mediator(s) on the association between BMI of the father and BMI of the child.

#### Intermediate effects

The effects of BMI of the father on M1 (PA/ST of the father) were both significant (a1-path; model 1 (PA); coefficient: -0.059, SE: 0.019, *p* = 0.002; model 2 (ST); coefficient: 2.238, SE:0.725, *p* = 0.002), indicating that a higher BMI of the father was associated with less PA and more ST of the father. Also the effects of M1 (PA/ST of the father) on M2 (PA/ST of the child) were both significant (a3-path; model 1 (PA); coefficient: 0.281, SE: 0.023, *p* < 0.001; model 2 (ST); coefficient: 0.345, SE: 0.025, *p* < 0.001), indicating that more PA and ST of the father was associated with more PA and less ST of the child, respectively. Furthermore, only in model 1 (PA), the effect of M1 (PA of the child) on BMI of the child was found significant (b2-path; coefficient: -0.050, SE: 0.022, *p* = 0.026), indicating that more PA of the child was related to a lower BMI of the child. All other effects in both models were found non-significant (i.e. model 1 (PA); a2-path, coefficient: 0.001, SE: 0.013, *p* = 0.967; b1-path, coefficient: 0.027, SE: 0.017, *p* = 0.107; model 2 (ST); a2-path, coefficient: 0.463, SE: 0.542, *p* = 0.393; b1-path, coefficient: 0.000, SE: 0.001, *p* = 0.922; b2-path, coefficient: 0.001, SE: 0.001, *p* = 0.103).

#### Indirect (mediational) effects

Only in model 1 (PA) a significant indirect (mediational) effect was found, i.e. the mediational effect of BMI of the father on BMI of the child via both M1 (PA of the father) and M2 (PA of the child) (M1&M2-path; coefficient: 0.001, 95% CI: [0.001, 0.002]). In both model 1 (PA) and model 2 (ST), all other indirect effects were found non-significant (model 1 (PA); a1xb1-path, coefficient: 0.000, 95% CI: [− 0.004, 0.003], a2xb2-path, coefficient: 0.000, 95% CI: [− 0.002, 0.002]; model 2 (ST); a1xb1-path, coefficient: 0.000, 95% CI: [− 0.002, 0.002], a2xb2-path, coefficient: 0.000, 95% CI: [− 0.001, 0.002], M1&M2-path, coefficient: 0.002, 95% CI: [0.000, 0.002]). As the direct effect (c’-path) of BMI of the father on BMI of the child was not reduced to zero in model 1 (PA) (i.e. the model with the significant mediational effect) (coefficient: 0.070, SE: 0.009, *p* < 0.001), this was only a partial mediation effect and effect sizes were only small (effect sizes_cz_ < 0.01).

## Discussion

The aim of the present study was to determine whether the association between fathers’ weight status and their children’s weight status is mediated by fathers’ and children’s movement behaviours (i.e. physical activity (PA) and screen time (ST)), in six European countries. With this, we aimed to gain more insight into the specific and unique influence that fathers have on their children’s weight status, and the specific pathways through which this influence occurs. In line with our hypothesis, the present study found that the association between weight status of the father and weight status of the child (partially) occurred through both the father’s and the child’s PA levels. This might, as suggested in previous research, imply that fathers play a unique and important role in establishing and maintaining positive PA habits in their children*,* thereby affecting their weight status [44, 45]. The significant association found between fathers’ PA and their children’s PA in the mediation analysis is consistent with previous research [22, 33]. Studies have indeed shown that fathers typically engage in more vigorous, active, risky and stimulating play with their children than mothers [46, 47], and are better role models for fundamental movement skills (e.g. catching, throwing) due to their increased opportunity and encouragement to learn and practice these skills throughout life [47, 48]. Furthermore, if confirmed in a longitudinal design or an intervention study, the results of the present study might indicate that the established association between fathers’ and children’s weight status is not entirely determined by non-modifiable factors (e.g. genes). This could have important implications for future lifestyle interventions, as they suggest that intervening by focusing on lifestyle behaviours (i.e. PA) of fathers can be a good and important strategy to influence the lifestyle behaviours and weight status of children, thereby preventing childhood obesity [49]. As such, a first approach could be to focus on the fathers’ PA in order to improve the child’s PA and consequently the child’s weight status. A systematic review summarizing the effectiveness of PA interventions for adult males showed that most of these interventions had positive effects on their PA outcomes [50]. However, none of these studies were designed to influence children’s PA through fathers’ PA or measured the impact of change in fathers’ PA on children’s PA or weight status, making it difficult to draw conclusions on the effectiveness of this approach on health and health-related outcomes of children. Another possible approach might be to focus on *both* members of the father-child dyad. A novel way to do so, might be through “co-PA” (i.e. engaging fathers and children together in PA). Although the mechanisms of co-PA are still unclear, some recent studies already provided a good indication that engaging fathers and children in co-PA can indeed lead to positive outcomes related to behaviour and health [51, 52]. More specifically, two programs have recently been developed specifically targeting fathers and children, aiming to help overweight fathers lose weight and establish positive health behaviors for their children [51, 52]. The results of these intervention studies showed that engaging fathers and children in co-PA increased (total) PA in both fathers and children, and positively influenced their weight. Furthermore, father-child co-PA also improved the father-child relationship and the social-emotional well-being of the child, which has also been mentioned in other research as a consequence of co-PA [46, 48, 53]. Taken together, co-PA appears as a potentially promising approach, that might act on both the direct and indirect effects between father and child BMI. Further research on co-PA is however needed to better understand its mechanism, and to determine whether focusing on co-PA is of added value and thus more effective than focusing separately on PA of the father and PA of the child. Moreover, a longitudinal design or an intervention study is warranted to confirm the findings discussed above, before definite conclusions and intervention recommendations can be made. As such, we are currently developing the “Run Daddy Run” intervention specifically targeting fathers and their children in order to improve their co-PA and limit their screen time.

In contrast to PA, the present study did not find a significant mediation effect of ST of the father and ST of the child on the association between BMI of the father and BMI of the child. Although we did found a significant positive association between fathers’ ST and their children’s ST -which is interesting as previous research on this association was currently scarce and inconclusive (33, 54, 55)-, a possible reason for the fact that no mediation effect was found might be that mainly mothers have an influence on their children’s ST, as previously suggested in research [54, 55]. Furthermore, another potential reason for the absent mediation effect of ST could be due to the non-significant association between ST of the child on BMI of the child. As significant associations between children’s ST and their weight status have been established in previous research [56–60], it might be that the strength of this association is underestimated in the present study, causing a non-significant mediation effect. Similarly, although a significant (partial) mediation effect of PA of the father and PA of the child on the association between weight status of fathers and weight status children was found in the present study, effect sizes were only small. Therefore, the clinical meaningfulness of the results should be interpreted with caution. The small effects might be due to the small association found between BMI and PA of the father. This is surprising as previous studies have shown stronger associations between adults’ weight status and their PA levels [61–63]. A reason for the weak or non-significant associations might be that BMI, PA and ST of the father was self-reported, and children’s PA and ST was based on parental report. Parental report is a subjective proxy-measure that may be biased. Using objectively measuring BMI, PA and SB may overcome this issue, which is therefore recommended for future research. Furthermore, two of our questions used for calculating the PA and ST measure (i.e. children’s PA on weekend days and fathers’ ST on weekday) had a lower reliability. Despite the fact that the reliability of the other questions measuring PA and ST questions was moderate to excellent, using existing validated and reliable questionnaires to measure PA and ST is recommended. Last, small effect sizes can also be due to residual confounders such as dietary behaviour, which was not accounted for in the present study.

Although the cross-sectional design of the study is a limitation, a strength of the present study is that standardized (paternal) data were included from six European countries, increasing the generalizability of the results and providing a large sample of fathers (*n* = 899). Although fathers represented only 10.1% of the total sample of the Feel4Diabetes-study (i.e. which is similar to previous research showing that mothers represent the majority of research participants [17]), our sample of fathers was still large enough to have a meaningful representation of fathers. Descriptive data from this study confirmed that our sample of fathers was indeed representative for the general population, with descriptive data (e.g. age, BMI, education level, etc.) similar to previous prevalence studies [64–66]. The underrepresentation of fathers in the Feel4Diabetes-study may be due to the fact that no recruitment strategies were used that explicitly targeted fathers. This may have caused a selection bias towards fathers who are generally more involved in child care and motivated for health and healthy lifestyle behaviours. Future research can target this issue by including recruitment strategies in which fathers are explicitly invited to participate (i.e. fathers often assume that the term “parent” is interchangeable with “mother” [16]), by targeting father-focused recruitment venues (e.g. work settings) and by clearly communicating the salient benefits and motivators for fathers (e.g. enhancing father-child relationship, spending quality time with their children) [16, 67].

## Conclusions

The present study showed that the association between fathers’ and children’s weight status is (partially) mediated by fathers’ and children’s PA (but not their ST). This helps us gaining more insight into the specific pathway through which fathers’ weight status influences their children’s weight status, and might imply that this association is not entirely determined by non-modifiable factors (e.g. genes). If confirmed in a longitudinal design or in an intervention study (e.g. the Run Daddy Run intervention), these findings might also have implications for future lifestyle interventions, as they indicate that intervening by focusing on fathers’ and children’s PA (i.e. preferably through engaging them together in PA) can be an important and novel strategy to influence weight status of children, thereby preventing childhood obesity.

## Data Availability

The data of the present study is available from the corresponding author on reasonable request.

## Abbreviations

BMI: Body Mass Index
PA: Physical activity
SB: Sedentary behaviour
ST: Screen time
